# Erratum to: Globally deployed sorghum aphid resistance gene *RMES1* is vulnerable to biotype shifts but is bolstered by *RMES2*

**DOI:** 10.1002/tpg2.20499

**Published:** 2024-07-29

**Authors:** Carl VanGessel, Brian Rice, Terry J. Felderhoff, Jean Rigaud Charles, Gael Pressoir, Vamsi Nalam*, Geoffrey P. Morris

This erratum corrects the following:

Vamsi Nalam should be listed as a co‐corresponding author.
